# ﻿New camaenid genus and species from Zhejiang, East China (Eupulmonata, Helicoidea)

**DOI:** 10.3897/zookeys.1202.118964

**Published:** 2024-05-15

**Authors:** Min Wu, Tian Chen, Wang Shen

**Affiliations:** 1 School of Life Sciences, Nanjing University, Xianlindadao 163, Qixia, Nanjing 210023, China Nanjing University Nanjing China; 2 Southern University of Science and Technology, Xueyandadao 1088, Nanshan, Shenzhen 518055, China Southern University of Science and Technology Shenzhen China

**Keywords:** Anatomy, Camaenidae, mantle lobe, molecular phylogenetics, new genus, new species, taxonomy

## Abstract

We report a new land snail species representing a new genus from the mountainous area of Zhejiang, China. The snail has a depressed shell with granules all over the surface. The soft part of the new taxon is characterized by the presence of a mantle lobe whose form is reviewed herein across a wide range of helicoid snails, the presence of a developed epiphallic papilla, and the absence of a penial sheath, a dart sac apparatus and a flagellum. As indicated by a molecular-based phylogeny (16S + ITS2), the new taxon is deeply nested in the eastern Asian camaenid genera and shows a close relationship with the camaenids distributed in Central China.

## ﻿Introduction

The first camaenid described in Zhejiang (= Dshè-dshiang, Chekiang [浙江]), China, was *Acustaravida* (Benson, 1842). So far, there are about 20 species of camaenid land snails known from Zhejiang, grouped into seven genera, namely *Acusta* Martens, 1860, *Aegista* Albers, 1850, *Bradybaena* Beck, 1837, *Nesiohelix* Kuroda & Emura, 1943, *Plectotropis* Martens, 1860, *Satsuma* A. Adams, 1868 and *Traumatophora* Ancey, 1887. *Acustaravida*, a widespread species in Central and East China (Möllendorff 1875, 1884; Gredler 1878; Heude 1882; Pilsbry 1888, 1934; Gude 1902b; Yen 1935, 1936, 1938, 1939, 1942, 1948; Hwang et al. 2021; etc.), is known from Zhoushan (= Chusan, Chowshan, the type locality [舟山]), Tonglu [桐庐], Moganshan [莫干山], Fuyang [富阳], Jiande [建德](= Yenchow [严州]), Lutzepu [?芦茨村], Xiaoshan (= Hsiaoshan [萧山]) of Zhejiang (Benson 1842; Möllendorff 1884, 1899; Gude 1902a, 1902c; Yen 1948) and the neighboring provinces (Möllendorff 1875, 1899; Yen 1936). The congener *A.redfieldi* (Pfeiffer, 1852), widespread in Central and South China (Heude 1882; Möllendorff 1884, 1899; Pilsbry 1888; Gude 1902a, 1902c; Yen 1938, 1939, 1942), was recorded with imprecise localities from Zhejiang (Möllendorff 1884, 1899).

*Bradybaenacremata* (Heude, 1882), may not be a Zhejiang member because its only locality Wuyuan (= Wu-yüan [婺源]) (Heude 1882; Möllendorff 1884; Pilsbry 1887; Yen 1939; Zilch 1968) belongs to Jiangxi [江西] but not mistakenly “Zhejiang” (Möllendorff 1884), which some authors subsequently followed (e.g., Gude 1902c). However, the distribution of this species in Zhejiang is possible because Wuyuan is close to West Zhejiang. A similar situation is also evident for *B.dichroa* (Pfeiffer, 1846), originally described from Shanghai [上海] (Möllendorff 1884; Pilsbry 1888; Yen 1942) neighboring northern Zhejiang, but treated as a species from Zhejiang (Gude 1902c). *Bradybaenasimilaris* (Rang, 1831), from Central China [including synonym *B.similarisnucleus* (Deshayes, 1873)] and Southeast China (Heude 1882; Möllendorff 1884; Gude 1902a, c; Jones and Preston 1904; Pilsbry and Hirase 1905; Yen 1936, 1939, 1942; Kuroda and Habe 1949; Zilch 1968, 1974) and “Nördlich von Shanghai scheint sie zu fehlen” (Möllendorff 1884), is also distributed in Zhejiang (Gude 1902c; Yen 1948). Interestingly, the distribution of *B.similaris* in northern China (e.g., Yen 1935) is possibly anthropogenic (personal observation by Wu M).

Considering that *Mastigeulotakiangsiensis* (Martens, 1875) is distributed in Central China including Hubei [湖北], Jiangxi, and Sichuan [四川], which are west of Hongzehu Lake [洪泽湖] of Anhui [安徽] (Heude 1882; Gredler 1884; Möllendorff 1884; Pilsbry 1934; Yen 1942; etc.), the distribution record of one of its subspecies *M.kiangsiensishilberi* (Kobelt, 1894) in Zhejiang (Gude 1902c) rather than Hubei (Hankow = Hankou [汉口] is the possible locality, Zilch 1968) is quite doubtful. Another species whose distribution was falsely recorded as Zhejiang is *Pseudiberustectumsinense* (Martens, 1873) (Gude 1902a), as all evidence shows that it is a species geographically limited to Jinan [济南] of Shandong [山东] (Martens 1873; Möllendorff 1884; Pilsbry 1888; Gude 1902c; Yen 1939, 1942; Zilch 1968; Wu and Qi 2006; Zhang et al. 2021).

*Aegistachinensis* (Philippi, 1845) was recorded from Moganshan, Fengshiu (= Fenshui [分水] of Tonglu) and Tonglu (Yen 1948). Four *Plectotropis* were found in Zhejiang. *Plectotropisbrevibarbis* (Pfeiffer, 1859) has the widest distribution as it is widespread in Wongkiang (= Oujiang [瓯江]), Fengshiu of the SE region (Yen 1948) and the NW region (Zhou et al. 2011) of the province or so-called “north of China”, possibly referring more to Zhejiang (Pfeiffer 1859; Pilsbry 1887) than northern China in the present sense. *Plectotropisbrevibarbis* is also known from Ningguo [宁国] of Anhui and the region between Shanghai and Wuyuan, both bordering northern Zhejiang (Heude 1882; Möllendorff 1884; Yen 1939). The species *P.sedentaria* (Heude, 1885), originally described from Fengjie [奉节] of Chongqing [重庆] (previously a part of Sichuan) (Heude 1885; Yen 1938) is present in Chiangshan [江山], Zhejiang (Yen, 1948). *Plectotropisbarbosella* (Heude, 1882), the third species of the genus, was reported from Shanghai, Taihu Lake [太湖], and a variety of localities in Zheijang (Heude 1882; Möllendorff 1884; Pilsbry 1888; Gude 1902c; Yen 1939, 1942,1948; Zilch 1968). *P.scitula* (Pilsbry & Hirase, 1908) was originally reported from Hangzhou [杭州] (Pilsbry and Hirase 1908). *Plectotropistrichotropistrichotropis* (Pfeiffer, 1850) was reported from Shanghai alongside northern Zhejiang, Wuyuan and Dongliu [东流] adjacent to western Zhejiang (Gredler 1881; Möllendorff 1884; Heude 1885; Pilsbry 1888; Yen 1942) and was listed as a species in Zhejiang by Gude (1902c) who similarly treated *P.trichotropislaciniata* (Heude, 1882) (Heude 1882; Pilsbry 1888; Gude 1902c; Yen 1939, 1942; Zilch 1968) as a species from Zhejiang. Another subspecies *P.trichotropisningpoensis* (Boettger, 1892) was found in eastern Zhejiang (Pilsbry 1892; Gude 1902c; Yen 1939; Zilch 1968).

Three or four *Satsuma* species, with only one verified for generic position, have been reported from Zhejiang. *Satsuma* (?) *fortunei* (Pfeiffer, 1850) has been reported from several localities in Zhejiang (Yen 1948), from which the species from Anhui and Shanghai, each bordering West and North Zhejiang, has been reported (Heude 1882; Pilsbry 1887). *Satsumalaeva* (Pilsbry & Hirase, 1908) was first described from Hangzhou (Pilsbry and Hirase 1908; = *Satsumauncopila* sensu Wang et al. 2014, see Zhang et al. 2019) and then Yen (1948) found it in nearby Tonglu and Lutzepu. *Satsuma* (?) *uncopila* (Heude, 1882), a species common in the Yangtze River valley (Heude 1882; Möllendorff 1884; Pilsbry 1887; Yen 1939, 1942; Zilch 1968; Zhang et al. 2019), was found in Moganshan at Southeast Anhui and South Jiangsu (Yen 1948). *Satsuma* (?) *latilabris* (Möllendorff, 1874) is distributed in Jiujiang [九江], Jiangxi (Möllendorff 1874, 1884; Zilch 1968; Zhang et al. 2019), which is located spatially near to West Zhejiang and was listed by Gude (1902c) as a member of Zhejiang.

*Cathaicafasciola* (Draparnaud, 1801), usually seen in horticulture environments, is a synanthropic species in Zhejiang (personal observation by Wu M).

With regard to the large-sized camaenids in the province, *Nesiohelixcecillei* (Philippi, 1849) is known from Tiantong (= Tien-tung or Tien-tong [天童]) of the east region (Möllendorff 1884; Pilsbry 1890; Gude 1902c; Yen 1939, 1942; Zilch 1968). *Nesiohelixmoreletiana* (Heude, 1882) is distributed in Hangzhou and Moganshan (Pilsbry 1890; Gude 1902c; Yen 1939, 1948; Habe 1945; Wu and Asami 2017), and in Ningde [宁德] of NE Fujian and Guangde [广德] of Anhui neighboring North Zhejiang (Heude 1882; Möllendorff 1884). *Nesiohelixyeni* Wu & Asami, 2017 was found sympatrically with *N.moreletiana* in Hangzhou (Wu and Asami 2017). Another large camaenid distributed in Zhejiang is *Traumatophoratriscalpta* (Martens, 1875), which is also known from Jiangxi (Boyanghu = Poyanghu [鄱阳湖], Fuzhou [抚州]), Hubei, and Zhejiang (Heude 1882; Gredler 1884; Möllendorff 1884; Pilsbry 1890; Gude 1902c; Yen 1939, 1942; Wu 2019). The fossils of *T.triscalpta*, along with those of *A.ravida*, *A.redfieldi*, and *B.similaris*, were found in the sedimentary outcrops of the Upper Pleistocene near Zhenjiang and Nanjing [南京] of Jiangsu [江苏], whose sites lie approximately 100 km north to North Zhejiang (Yen 1943).

In contrast to the northern parts of East China, more than 74% of the mountainous area in Zhejiang provides a considerable variety of microhabitats where speciation of land snails is expected. We have recently found a camaenid slightly smaller than *Nesiohelixmoreletiana* and *N.yeni* in shell size that represents an unknown camaenid in terms of genital anatomy and molecular systematics. The presence of a lobe on the mantle collar in this new snail impels us to compare this body part in a wider range of helicoid snails.

## ﻿Materials and methods

### ﻿Morphology

Living specimens were relaxed by drowning in water and then fixed in 70% ethanol. The shell and genital system were measured with digital vernier calipers and from photograph to the nearest 0.1 mm, respectively. Whorl number was counted as described by Kerney and Cameron (1979), with 0.125 (= ^1^/_8_) whorls accuracy. Parts of the genital system were measured after the specimens were sufficiently fixed in 70% ethanol. Radula preparation: The buccal mass was removed and treated in 10% sodium hydroxide solution below 60 °C for up to 10 min before the radula was extracted. The released radula was cleaned with water using a sonic cleaner and then transferred into 75% ethanol before being mounted. The radula was examined under a scanning electron microscope (SEM; Sigma 500). To observe the possible lobe near the mantle edge, the hardened mucus and small pieces of dirt were carefully removed with a soft brush under water. Directions used in descriptions: proximal = towards the genital atrium; distal = away from the genital atrium.

The Chinese name for the person, new taxon or locality is provided only once in square brackets when necessary.

### ﻿Molecular phylogenetic analyses

Whole genomic DNA was extracted from a piece of pedal muscle of the ethanol-preserved specimens using TIANamp Marine Animals DNA Kit. Each 25 μL PCR mixture consisted of 12.5 μL cwbio 2× Es Taq MasterMix Dye, 9.5 μL ddH_2_O, 1 μL template DNA, 1 μL forward primer (10 μL/L) and 1 μL reverse primer (10 μL/L). Primers used for ITS2 were LSU1: 5’-CTAGCTGCGAGAATTAATGTGA-3’, LSU3: 5’-ACTTTCCCTCACGGTACTTG-3’ (Wade and Mordan 2000), for 16S were 16SAR: 5’-CGCCTGTTTATCAAAAACAT-3’, 16SBR: 5’-CCGGTCTGAACTCAGATCACGT-3’ (Palumbi et al. 1991). The conditions for thermal cycling, performed on an Eastwin ETC811 Cycler, for 16S were 60 s at 96 °C for pre-denaturing, 35 cycles of 30 s at 94 °C, 30 s at 55 °C and 60 s at 72 °C, and a final extension at 72 °C for 10 min; for ITS2 4 min at 94 °C for pre-denaturing, 30 cycles of 20 s at 94 °C, 20 s at 55 °C and 40 s at 72 °C, and a final extension at 72 °C for 10 min. The amplicons were examined on a 1% agarose gel for quality and fragment size, then were purified and sequenced on an automated sequencer.

Chromatographs and sequences were studied and compiled in Sequencher v.4.5. For phylogenetic analysis, sequences of the new taxon (GenBank accession numbers: 16S, OR209732; ITS2, OR209722) and all those from a recently published work (see Wu et al. 2023, table 3) were included. The alignment of ITS2 and 16S was performed in batches with MAFFT v.7.505 (Katoh and Standley 2013) in PhyloSuite v.1.2.3 (Zhang et al. 2020; Xiang et al. 2023) using ‘--auto’ strategy and normal alignment mode. Gap sites of rRNA genes were removed with trimAl v.1.2rev57 (Capella-Gutiérrez et al. 2009) using “-automated1” command. DAMBE v.7.3.32 (Xia 2018) was employed to make the saturation tests. Unsaturated sequences were concatenated in the same order for subsequent analyses. The best-fit partition model (Edge-linked) was selected under the BIC criterion using ModelFinder (Kalyaanamoorthy et al. 2017). Bayesian inference phylogenies were inferred using MrBayes v.3.2.7a (Ronquist et al. 2012) under a partition model (2 parallel runs, 800,000 generations), in which the initial 25% of sampled data were discarded as burn-in. Maximum-likelihood phylogenies were inferred using IQ-TREE (Nguyen et al. 2015) under the Edge-linked partition model for 5000 ultra-fast (Minh et al. 2013) bootstraps, as well as the Shimodaira-Hasegawa-like approximate likelihood-ratio test (Guindon et al. 2010).

### ﻿Abbreviations

**At** – atrium; **BC** –bursa copulatrix; **BCD** – bursa copulatrix duct; **Ep** – epiphallus; **EpP** – epiphallic papilla; **FO** – free oviduct; **P** – penis; **PR** – penial retractor muscle; **Va** – vagina; **VD** – vas deferens.

### ﻿Depositories

**HBUMM** mollusc collection of the Museum of Hebei University, Baoding, China

**IZCAS** Zoological Museum, Institute of Zoology, Chinese Academy of Sciences, Beijing, China

## ﻿Results

### ﻿Phylogeny of the studied taxa

A concatenated matrix of 80 terminals (including outgroup) × 1045 bp (296 bp from partial 16S sequence and 749 bp from partial ITS2 sequence) was used in the subsequent analyses. Both 16S and ITS2 were unsaturated. For the Bayesian method, GTR+F+I+G4 was selected as the best evolution model for 16S, as well as K2P+G4 for ITS2. For the ML method, GTR+F+I+G4 was the best model for 16S I and K2P+I+I+R2 was the best model for ITS2.

The obtained phylograms using Bayesian inference and the maximum-likelihood analysis are topologically similar to each other except for two positions, as indicated in Fig. 7. Most clades received high support. The genera *Nesiohelix* Kuroda & Emura, 1943, *Traumatophora* Ancey, 1887, *Camaenella* Pilsbry, 1893, *Aegista*, *Plectotropis* Martens, 1860 are basal on the tree. *Sinocamaena* gen. nov. is inside the clade consisting of a variety of taxa that are almost exclusively endemic to the Southern Gansu Plateau. The phylogram topologically agrees well with that of Wu et al. (2023).

### ﻿Comparative study of the mantle lobe in helicoids, especially for eastern Asian taxa

In some preserved specimens, the mantle lobe is covered with curdled mucus and is difficult to observe. At the mantle collar, the lobe-like flesh (literally indistinguishable from “lobe”, e.g., in Chen et al. 2021) near the anus+ pneumostome, forms a more or less developed elongating part of the structure of the anus orifice, i.e., the suprapneumostomal and subpneumostomal lobes (e.g., in *Napaeusnanodes*, fig. 6A in Henríquez et al. 1993). In this work, the mantle lobe refers to a single separate piece of fleshy lamina (e.g., Fig. 3B) on the opposite side of the lobe-like structure near the anus + pneumostome (e.g., Fig. 3A), which appears as a thin fleshy flap that is usually attached to the inner wall of the mantle collar. If present, the mantle lobe of a dextral-shelled snail is on the left side, and in the case of a sinistral-shelled snail on the right side of the mantle collar.

We observed mantle lobes in the voucher specimens phylogenetically investigated here and in other specimens including *Acusta*, *Aegista*, *Bradybaena*, *Camaena* Albers, 1850, *Camaenella*, *Euhadra* Pilsbry, 1890, *Nesiohelix*, *Plectotropis* Martens, 1860, *Satsuma*, *Sinocamaena* gen. nov. (see below), and *Traumatophora* (Table 1).

The groups that do not have a mantle lobe are the following: “*Bradybaena*” in Central China, *Buliminidius* Heude, 1890, *Cathaica* Möllendorff, 1884, *Fruticicola* Held, 1837, *Metodontia* Möllendorff, 1886, *Pseudiberus* Ancey, 1887, *Pseudobuliminus* Gredler, 1886, *Stilpnodiscus* Möllendorff, 1899, and *Trichobradybaena* Wu & Guo, 2003 (Table 1).

**Table 1:** Material examined for the comparative morphology of the mantle lobe in some helicoids.

Species	Collection information of the examined specimens
*Acustaravida* (Benson, 1842)	HBUMM06242, Xiuning, Anhui, China, coll. Zheng W, Liu JY, 2007-V-19
*Aegistachinensis* (Philippi, 1845)	HBUMM06481, Nanshan, Zhenjiang, China, coll. Wu M & Xu Q, 2011-VI-12
Angiomphalia (Angiomphalia) guljaensis Wu, 2004	HBUMM05177, Gulja, Xinjiang, China, coll. Wu M, Ayken A, 2002-VI
*Bradybaenabrevispira* (H. Adams, 1870)	HBUMM04167, Baidicheng, Fengjie, China, coll. Wu M, 2004-VII-20
*Bradybaenacircula* (Pfeiffer, 1846)	HBUMM06859, Yoron-to Island, Japan. Individual, No. 3, coll. Hou L & Asami T, 2012-VI
“*Bradybaena*” *eris pachychila* (Möllendorff, 1899)	HBUMM05493, Wenxian, Gansu, coll. Wu M et al., 2006-IX-28
*Bradybaenalinjun* Wu & Chen, 2019	HBUMM08241, types
*Bradybaenaqixiaensis* Wu & Asami, 2017	HBUMM06841, paratypes, Qixiashan, Nanjing, China, coll. Wu M, Fang YX & Wang DB, 2012-6-26
*Bradybaenasimilaris* (Rang, 1831)	HBUMM006861, Matsumoto, Nagano, Japan. No. 3; coll. Hou L & Asami T, 2012-VI-13
“*Bradybaena*” *strictotaenia* (Möllendorff, 1899)	HBUMM05467, Wenxian, Gansu, China, coll. Wu M, Liu JM, Zheng W and Gao LH, 2006-IX-27
“*Buliminidius*” *achatininus* (Möllendorff, 1899)	HBUMM00523, Gansu, China
“*Buliminidius*” *hirsutus* (Möllendorff, 1899)	HBUMM06565, Jiuzhaigou, Sichuan, coll. Wu, Xu & Buhda, 2011-VIII-14
*Camaenamenglunensis* Chen & Zhang, 1999	HBUMM01701, Bubang, Yunnan, China, coll. Wu M & Wiktor A, 2002-VII-25
*Camaenellaplatyodon* (L. Pfeiffer, 1846)	HBUMM08457, Hainan, China, 2020-IX
*Cathaicabuvigneri* (Deshayes, 1873)	HBUMM08140, Huanxian, Gansu, China, coll. Sheng XF et al., 2017-VII-29
*Cathaicafasciola* (Draparnaud, 1801)	HBUMM08144, Qingyang, Gansu, colln. Sheng XF et al., 2017-VII-28
“*Cathaica*” *gansuica* Möllendorff, 1899	HBUMM05666, Dangchang, Gansu, China, coll. Liu JM, Zheng W, 2006-X-02
“*Cathaica*” *pulveratricula* (Martens, 1882)	HBUMM08208, Dingxi, Gansu, coll. Sheng XF et al., 2017-VIII-4
*Cepaeahortensis* (Müller, 1774)	HBUMM05979, Slagelse, Denmark, coll. Guo JY, 2003-V-7–8
*Eueuhadra* sp.	HBUMM03341, Heishui, Sichuan, coll. Zhou HZ, 2001-VII-24–26
*Euhadraamaliae* (Kobelt, 1875)	HBUMM06851, Kyoto, Japan, No. 3, coll. Asami T, 2010; HBUMM06868–Kyoto, Japan, No. 2, coll. Asami T, 2010
*Euhadrasandaicommunis* Pilsbry, 1928	HBUMM06856, Kyoto, Japan, coll. Asami T
*Fruticicolafruticum* (Müller, 1774)	HBUMM01006, Lower Silesia Reserve Muszkowicki Las Bukowy near Henry Kow, Poland, coll. Wu M & Wiktor A, 1999-VI-26
*Helixpomatia* Linnaeus, 1758	HBUMM05972a, Nyborg, Denmark. coll. Guo JY, 2003-V-15
*Karaftohelixmiddendorffi* (Gerstfeldt, 1859)	HBUMM05924, Cypancebua, Russia, coll. Sayenko EM, 2002-IX-27
*Laeocathaica* spp.	All known species, see Wu et al. 2023
*Mastigeulotakiangsiensis* (Martens, 1875)	HBUMM04190, Badong, Hubei, China, coll. Wu M, Wu Q, Qi G, 2004-VIII-1
*Metodontiawenxianensis* Chen & Zhang, 2004	HBUMM03335, Wenxian, Gansu, China, coll. Chen DN & Zhang GQ, 1998-V-17 (synonym: *Metodontiabidentatus* Wu & Prozorova, 2006)
*Nesiohelixmoreletiana* (Heude, 1882)	HBUMM00054, Hangzhou, Zhejiang, China, coll. Chen DN, 1979-VIII-12
*Nesiohelix* sp.	HBUMM06873, Jiahe, Hunan, China, coll. Liu ZP, 2016-V-29
*Plectotropissterilis* (Heude, 1890)	HBUMM04568, Badong, Hubei, China, coll. Wu M, 2003-VIII-20
Ponsadenia (Mesasiata) duplocincta (Martens, 1879)	HBUMM05886, Xinyuan, Xinjiang, China, coll. Wu M, Ayken A, 2002-VI-05
*Pseudiberusliuae* Wu, 2017	HBUMM06759, Shijiba, Wenxian, Gansu, China, types, coll. Wu M, Xu Q & Buhda P, 2011-VI-10
“*Pseudobuliminus*” *piligerus* (Möllendorff, 1899)	HBUMM05428, Wenxian, Gansu, China, coll. Wu M et al., 2006-IX-27
*Pseudobuliminusstrigatus* (Möllendorff, 1899)	HBUMM05773, Wenxian, Gansu, China, coll. Wu M, 2006-IX-28
*Pseudobuliminussubcylindricus* (Möllendorff, 1899)	HBUMM04457, Wenxian, Gansu, coll. Chen DN & Zhang GQ, 1998-IV-27
*Pseudostegoderaqiului* Chen, 2021	IZCAS TM206978, holotype
*Satsumaguandi* Zhang, Zhu & Lyu, 2019	HBUMM08239, Wenyuan, Shaoguan, Guangdong, China, coll. Yu D
*Satsumauncopila* (Heude, 1882)	HBUMM03296, Zhongshanling, Nanjing, China, coll. Wu M, 2000-V-1. HBUMM06839, Tangshan, Nanjing, China, coll. Xu Q, Wang SY & Hou L, 2012-VI-29
*Sinochloritislii* Wu & Chen, 2019	HBUMM08294, types
*Sinochloritis* sp.	HBUMM04525, Lushui, Yunnan, coll. Chen DN, 1981-VI-5
“*Stilpnodiscus*” *entochilus* Möllendorff, 1899	HBUMM00076, Jiuzhaigou, Sichuan, China, coll. Chen DN & Zhang GQ, 1998-V-18
*Traumatophoratriscalpta* (Martens, 1875)	HBUMM06875, Tianmushan, Zhejiang, China, coll. Zhou DK, 2016-V
*Trichobradybaenasubmissa* (Deshayes, 1873)	HBUMM01504, Meitan, Guizhou, China, coll. Wu M, 2003-VIII-2

Other groups of helicoids from Camaenidae, Hygromiidae and Helicidae, which are not included in the present phylogenetic study but have been anatomically examined, have mantle lobes as in Hygromiidae: *Angiomphalia* Schileyko, 1978; in Camaenidae: *Eueuhadra* Wu, 2004, *Mastigeulota* Pilsbry, 1894, *Ponsadenia* Schileyko, 1978, *Pseudostegodera* Wu & Chen, 2021, *Sinochloritis* Wu & Chen, 2019 (Wu et al. 2019b) and *Sinorachis* Wu & Chen, 2019 (Wu et al. 2019a); and in Helicidae: *Cepaea* Held, 1837 and *Helix* Linnaeus, 1758 (Table 1).

### ﻿Systematics

**Helicoidea Rafinesque, 1815**

**Camaenidae Pilsbry, 1895**

#### ﻿ Sinocamaena

Taxon classificationAnimaliaEupulmonataCamaenidae

Wu gen. nov.

378D227E-76CA-5AB9-A639-0211CB665B24

https://zoobank.org/40C4A6B6-BC44-4F77-87E3-1DDF85815DB3

##### Chinese name.

中华坚螺属.

##### Type species.

*Sinocamaenacheni* Wu, gen. et sp. nov.

##### Diagnosis.

Shell depressed. Protoconch and teleoconch granulate. Protoconch strongly sculptured. Peristome expanded. Head wart low and tiny. Between the ommatophore insertions, a gland pore present. A mantle lobe present. Penial sheath absent. Epiphallus very short. Epiphallic papilla well developed. Flagellum absent.

##### Description.

Shell depressed. Whorls slightly convex. Suture slightly impressed. Umbilicus broad. Protoconch with granules on strong radial sculpture. Peristome expanded. Adult shell surface without ribs, hairs or scales. Growth lines fine and evenly broken into granules on teleoconch. Shell with several thin bands above and beneath carina.

General anatomy. A small pore externally present between ommatophore insertions. Eversible head wart very weakly developed. A mantle lobe present.

Genitalia. Penial sheath absent. Penis externally without penial caecum. Pilasters inside penis low and weak. Epiphallus very short. Epiphallic papilla rather developed. Flagellum absent.

##### Etymology.

This new genus is named after “sino” (= China) and “camaena” which is a camaenid genus that includes many large-sized helicoid species.

##### Distribution.

China: Zhejiang.

##### Remarks.

The new genus is conchologically close to many camaenids, such as *Camaena* Albers, 1850 and *Burmochloritis* Godwin-Austen, 1920, in having a large helicoid shell with multiple slender bands. In comparison to *Camaena* (sensu Schileyko 2003), the new genus has a strongly sculptured protoconch and an extremely short epiphallus (the part between penial retractor muscle insertion and vas deferens insertion), but has neither the axial corrugated pilasters within penis nor the flagellum. The new genus differs from *Burmochloritis* (Páll-Gergely et al. 2023) in the absence of the flagellum, the long cylindrical epiphallus, the penial caecum and the dart sac. *Sinocamaena* gen. nov. shares with *Sinochloritis* the possession of granules on the protoconch and the characters from both general and genital anatomy, including the presence of a visible gland pore between the ommatophore insertions, a mantle lobe, a well-developed epiphallic papilla and the absence of a penial sheath and a dart sac apparatus. Compared to *Sinochloritis*, the new taxon has neither a flagellum nor a long epiphallus with the cylindrical trunk, nor prominent penial pilasters. In terms of shell morphology, generally, the new genus looks different from any other Chinese indigenous camaenid genus. Regardless of the shell morphology, all the other Chinese camaenid genera having no dart sac apparatus, i.e., *Amphidromus* Albers, 1850, *Landouria* Godwin-Austen, 1918, *Pancala* Kuroda & Habe, 1949, *Satsuma*, *Yakuchloritis* Habe, 1955, *Pseudostegodera* and the above mentioned *Sinochloritis*, possess a well-developed flagellum in the male part of genitalia (table 1 in Wu 2023). The present phylogeny (Fig. 7) suggests that the new taxon is possibly the nearest relative of almost all the members of dart sac-bearing bradybaenines endemic to the South Gansu Plateau or Central China.

It is noteworthy that the taxa that are basal on the phylogram, i.e., *Nesiohelix*, *Traumatophora*, *Acusta*, *Bradybaena*, *Plectotropis*, *Aegista*, *Euhadra*, *Satsuma*, *Camaena*, have mantle lobes. In general, the camaenids from Central China constitute a monophyly that receives high support values (clade X in Fig. 7), in which all terminals have a dart sac apparatus but lack a mantle lobe. The extended study of mantle lobes in helicoids taken here suggests that the presence of the mantle lobe could be a widely distributed and possibly a plesiomorphic character in the superfamily Helicoidea.

#### ﻿ Sinocamaena cheni

Taxon classificationAnimaliaEupulmonataCamaenidae

Wu, gen. et sp. nov.

FA71C0AD-EEA6-54D5-A57C-39A6A8C7C2F5

https://zoobank.org/BAD768FA-1757-4938-83A2-E84FCE83B407

##### Chinese name.

陈氏中华坚螺.

##### Type material.

***Holotype***: a fully mature living snail, HBUMM08381-spec. 1, Zhangjiadi [张家地], Yunhe County [云和县], Lishui [丽水], Zhejiang Province; around oaks in remote forest, 27.974°N, 119.379°E, c. 820 m a.s.l., 2019-VIII, coll. Chen, Tian [陈天]; molecular voucher specimen HBUMM08381a. ***Paratypes***: five fully mature empty shells, HBUMM08381-spec. 2–6, same collection data as holotype; HBUMM08367, a fully mature living snail; Zhangjiadi, Yunhe County, Lishui, Zhejiang Province; oak woods, coll. Chen, Tian; molecular voucher specimen HBUMM08367a-1; HBUMM08382-spec. 1, a living snail that reared to maturity at laboratory, same collection data as holotype; a fully mature empty shell, HBUMM08370-spec. 1, Mihougu [猕猴谷], Fengyangshan [凤阳山], Longquan County [龙泉县], Lishui, Zhejiang Province; 27.897°N, 119.159°E, 1100 m a.s.l., 2019-VIII-26, coll. Ye, Shi-Han [叶诗涵].

##### Measurement of holotype.

Shell height 19.2 mm, shell breadth 44.3 mm, aperture height 15.9 mm, aperture breadth 22.2 mm, embryonic shell whorls 1^1^/_4_, whorls 4^5^/_8_.

##### Description.

**Figure 1.**
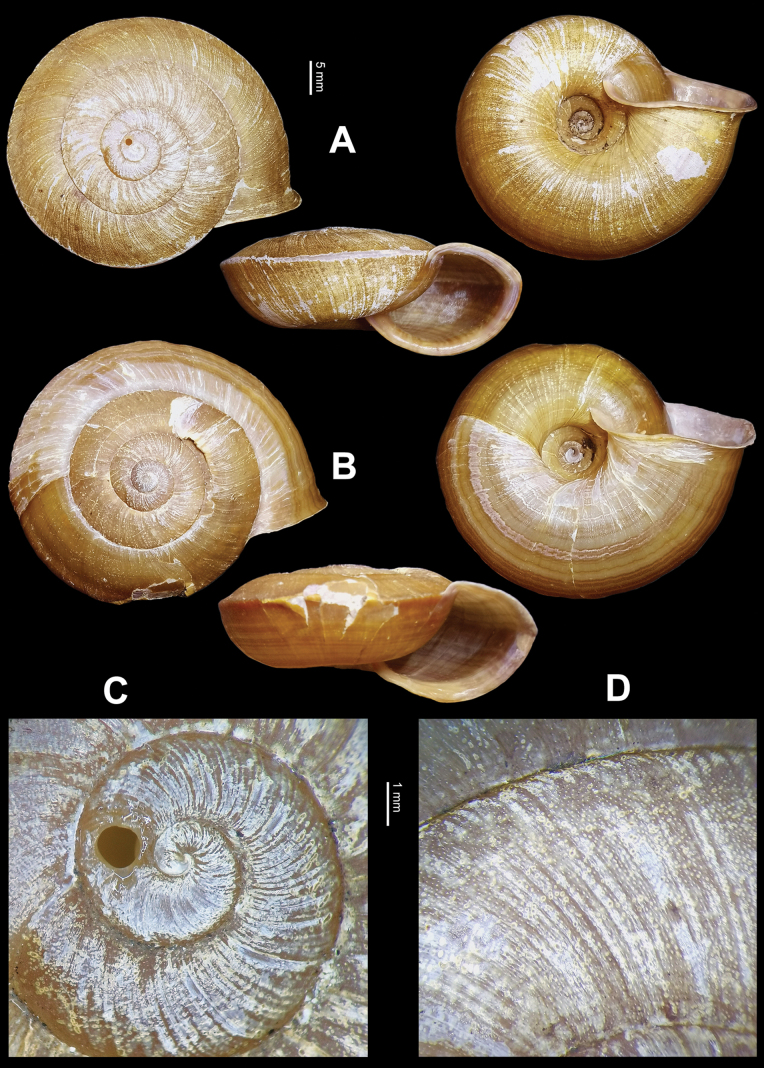
*Sinocamaenacheni* Wu, gen. et sp. nov. **A** holotype, HBUMM08381-spec. 1 **B** paratype, HBUMM08382-spec. 1, reared to maturity in laboratory **C, D** HBUMM08381, holotype **C** shell apex **D** shell surface. Upper scale for **A, B**; lower scale for **C, D**.

Shell (Fig. 1) large, depressed. Whorls slightly convex. Suture shallowly impressed. Umbilicus broad with embryonic whorls visible, approximately one-fifth of shell major diameter. Bottom-umbilicus transition changed gently. Columella oblique or obliquely curved. Columellar lip slightly covering umbilicus. Protoconch evenly granulate with strong radial sculpture (Fig. 1C). Teleoconch granulate, without hairs, scales or spiral furrows (Fig. 1D). Peristome evenly expanded, minutely sinuate. Aperture oblique, slightly expanded. Body whorl large, straight in front, sharply carinate above periphery. Aperture without inner ring-like thickening. Peristome thin, in faint purple. Shell dull and brownish yellow, with many clear thin brown bands above and beneath carina. Measurements (types, *N* = 8): shell height 17.5–21.0 mm (18.7 ± 1.15 mm), shell breadth 41.2–46.8 mm (43.9 ± 1.89 mm), aperture height 14.2–16.8 mm (15.4 ± 0.86 mm), aperture breadth 20.6–23.8 mm (22.3 ± 1.34 mm), embryonic shell whorls 1–1^1^/_4_ (1.125 ± 0.1157 whorls), whorls 4^3^/_8_–4^5^/_8_ (4.531 ± 0.1108 whorls), shell height/breadth ratio 0.41–0.45 (0.43 ± 0.013).

**Figure 2.**
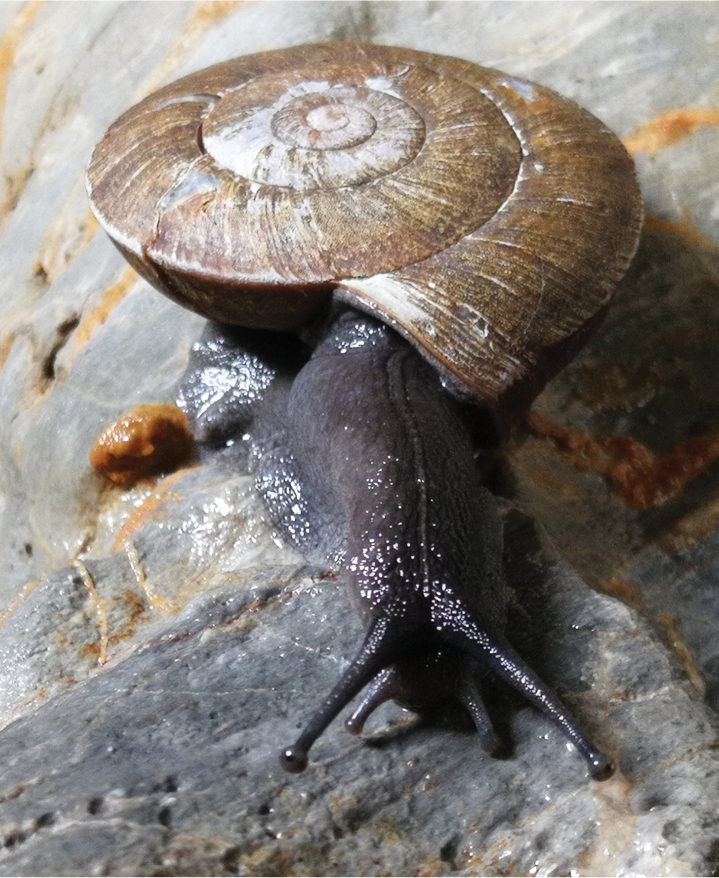
*Sinocamaenacheni* Wu, gen. et sp. nov., paratype, HBUMM08382-spec. 1, a specimen reared to maturity in laboratory.

**Figure 3.**
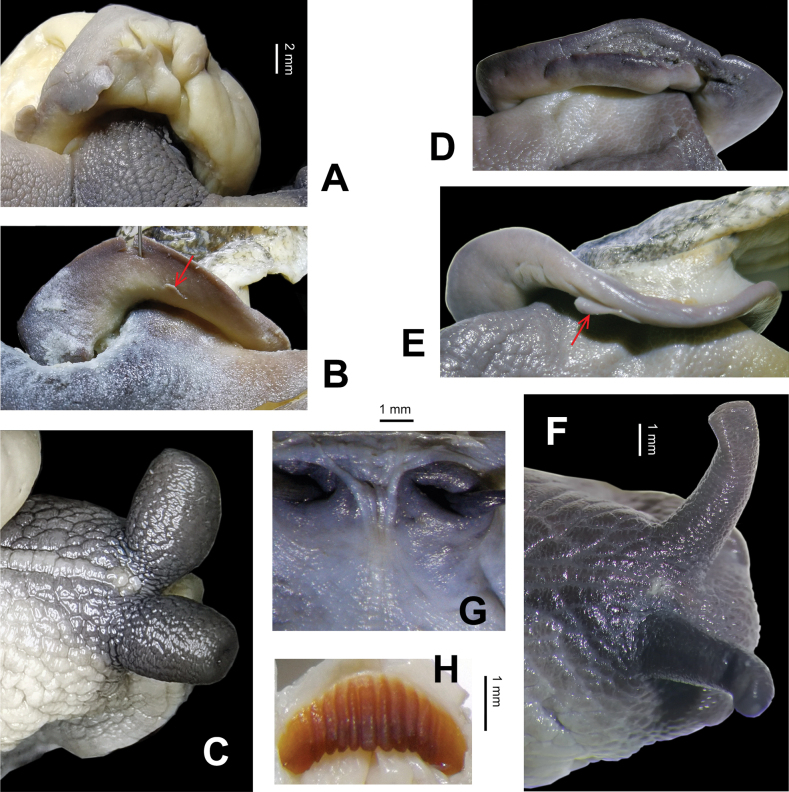
*Sinocamaenacheni* Wu, gen. et sp. nov., general anatomy **A–C** holotype, HBUMM08381-spec.1 **A** lobe-like structure near the anus+ pneumostome **B** mantle lobe on the left margin of mantle collar **C** anterior part of the animal, dorsal view, between ommatophore tentacles, showing the head gland where the pore/opening is not obvious **D–H** paratype, HBUMM08367-spec.1 **D** lobe-like structure near the anus+ pneumostome **E** mantle lobe on the left margin of mantle collar **F** dorsal view of anterior part of the animal, showing the pore among the contractive head gland **G** internal body wall of head, showing the head gland pore between the ommatophore tentacles **H** jaw, with basal muscle tissue remaining.

**Figure 4.**
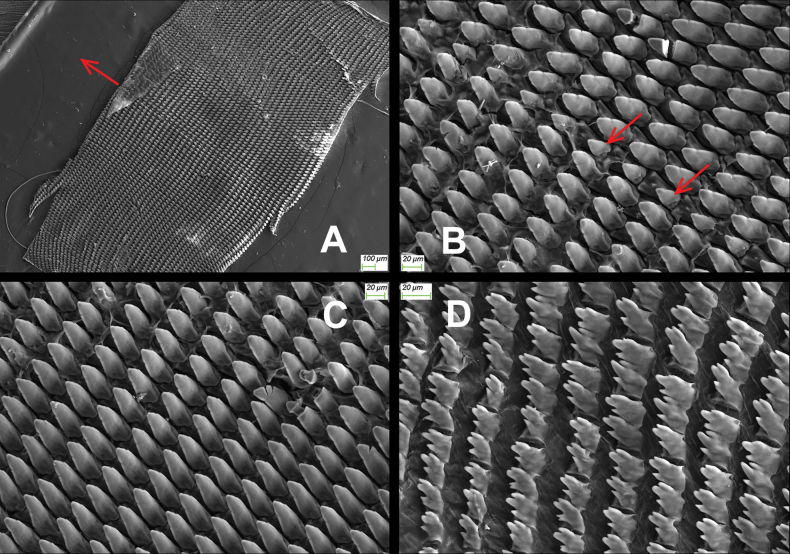
*Sinocamaenacheni* Wu, gen. et sp. nov. SEM photos. Paratype, HBUMM08367-spec.1 **A** section of radula, symmetry axis of radula arrowed **B** central teeth and lateral teeth; central teeth arrowed **C** lateral teeth **D** marginal teeth.

General anatomy (Figs 2–4). Externally, a small pore present between ommatophore insertions. Eversible head wart surrounding the pore very weakly present. A mantle lobe present. Tentacles and body in dark leaden-black; sole in color lighter than dorsal side. Jaw arcuate; with 12 more or less projecting ribs (Fig. 3H). Radula (HBUMM08367-spec. 1) comprises numerous transverse rows of teeth, each row containing approximately 131 teeth, 38+27+1+27+38. Central tooth symmetrically conic, without cuspid. Lateral tooth about more than two times larger than central tooth, strong conic medially, weakly uni-cuspid at both sides. Marginal teeth gradually changing from broadly tri-cuspid to tetra-cuspid (Fig. 4).

**Figure 5.**
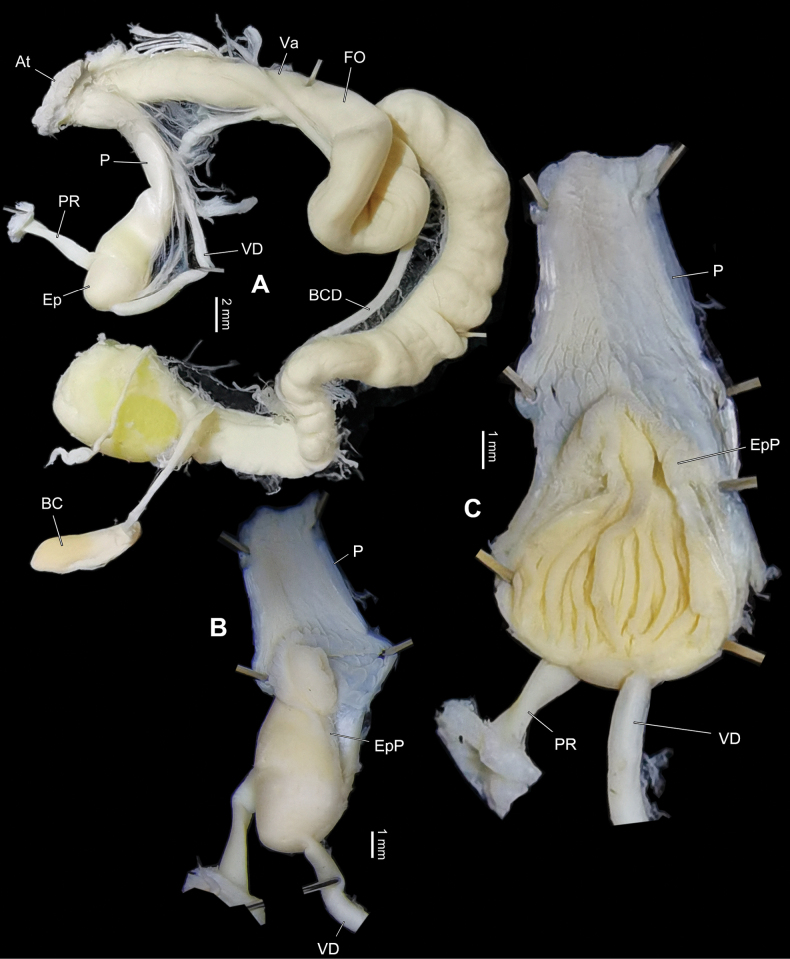
*Sinocamaenacheni* Wu, gen. et sp. nov., genitalia, holotype, HBUMM08381-spec.1 **A** general view of genitalia **B** exposed penis **C** exposed penial papilla.

**Figure 6.**
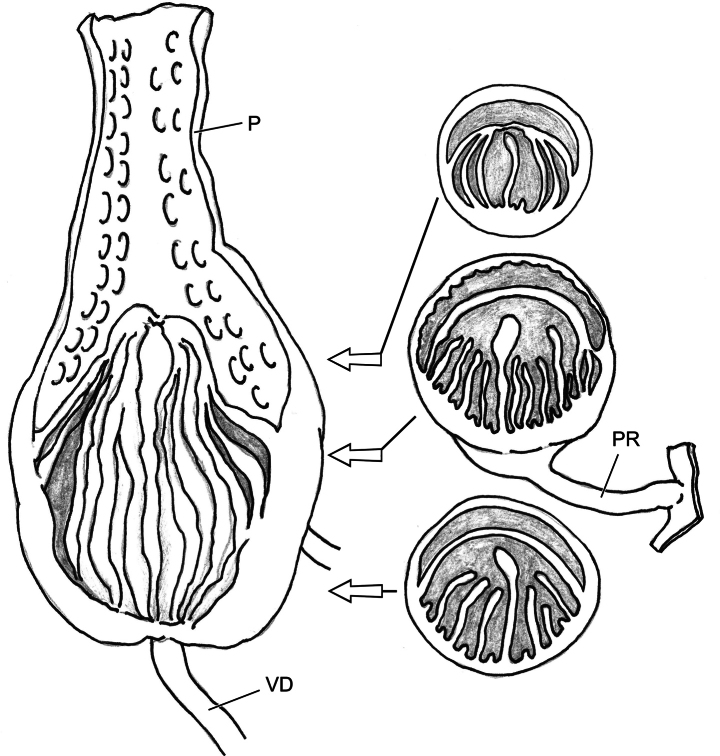
*Sinocamaenacheni* Wu, gen. et sp. nov., holotype, HBUMM08381-spec.1. exposed penis and exposed penial papillae, showing cross-sections.

Genitalia (Figs 5, 6). Penial sheath absent. Penis club-shaped, swollen near insertion of penial retractor muscle. Penis externally simple, internally with numerous longitudinal arranged low projections like scales, which do not connect to each other into pilasters along the penial inner wall. Epiphallic papilla rather developed, on side of penial retractor muscle insertion with about several longitudinal pilasters which join apically. Epiphallus very short and stout, internally with a septum longitudinally dividing epiphallus into two separate chambers which one is empty and another one with about 10 pieces of high and low pilasters among which middle one is the strongest (Fig. 6). Flagellum absent. Membranous sac surrounding terminal genitalia absent. Dart sac apparatus absent. Vas deferens thin, slightly thickened near epiphallus. Vagina thick, subequal to penis in length. Bursa copulatrix duct thin, proximally not expanded. Measurement in holotype: P – 9.9 mm; Ep – 2.4 mm; VD – 19.4 mm; PR – 4.8 mm; Va – 7.2 mm; FO – 5.4 mm; BC plus BCD – 55.3 mm.

##### Ecology.

This species was found in the litter layer in broad-leaved forest where oaks dominate (Fig. 8). However, the rediscovery of this species failed at one of the known localities (Zhangjiadi, Lishui) in April 2023.

##### Etymology.

This new species is named in memory of Professor Chen De-Niu [陈德牛 Nov 1939 – March 2024], a known malacologist working on Chinese land molluscs. Prof. Chen was one of the doctoral supervisors for Wu M.

##### Distribution.

Zhejiang (only from type localities: Yunhe, Longquan).

##### Remarks.

The new species and *Camaenavulpis* (Gredler, 1887) are superficially similar in having the densely and minutely granulate surface, numerous spiral thin bands and the general shape of shell. However, besides possessing a distinctly larger shell and a higher spire, the latter species has a totally different genital system, which has a long flagellum (HBUMM08664, Liannan [连南], Nature Reserve of Giant Salamander, Guangdong, China, 2023-VII, coll. Wang Chong-Rui [王崇瑞], Chen Hui [陈辉]). The new species can be promptly distinguished from all the other Chinese camaenid taxa in genitalia because of the absence of the dart sac apparatus and the flagellum.

**Figure 7.**
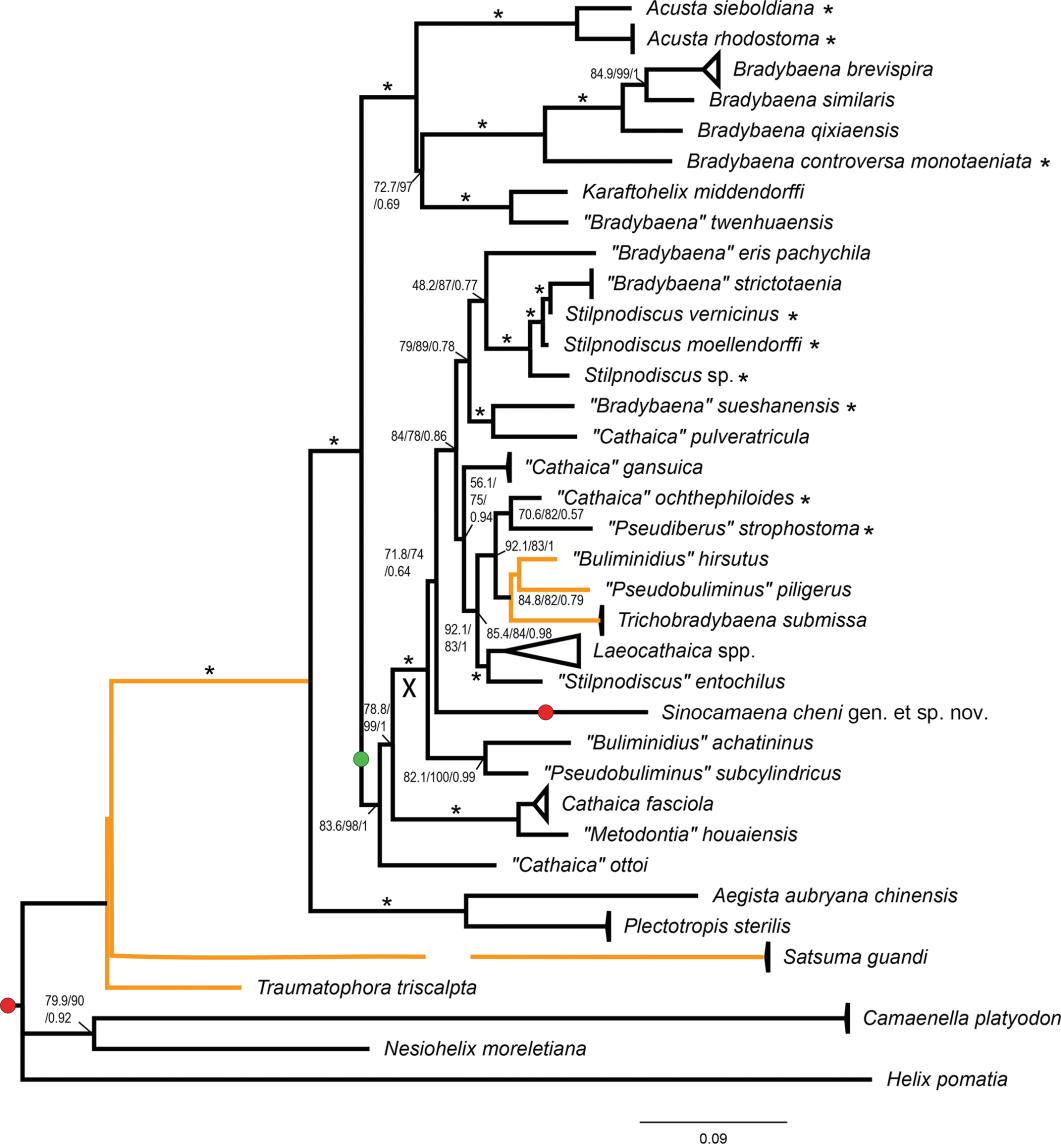
Maximum-likelihood phylogram based on the concatenated partial mitochondrial 16S and partial ITS2 sequences of East Asian camaenid species. The tree is rooted on *Helixpomatia*. Numbers near nodes indicate the Shimodaira and Hasegawa-approximate likelihood-ratios (SH-aLRT)/approximate Bayes test (aBayes)/ultra-fast bootstrap (IQ-TREE, maximum likelihood)/posterior probability (MrBayes, Bayesian inference). An asterisk on the branch indicates a clade with all well-supported values (SH-aLRT ≥ 80%, aBayes ≥ 0.95, BS ≥ 95%, PP ≥ 0.95). The broken branch indicates that the branch is shortened to exactly 1/2 the original length. Scale bar is for substitutions per site. Orange branches indicate where the BI tree topologically differs from the ML tree. Red dot/green dot indicates every terminal on that branch that has/has not a mantle lobe. An asterisk after the species name indicates that observation of the mantle lobe failed due to the bad condition of the specimen. The species under the genera in quotes are thought to be questionable generic assignments (Wu et al. 2023).

The phylogenetic analysis suggested that the new species/genus and the taxa distributed in Central China are possibly close relatives (Fig. 7). For other comments see the genus.

**Figure 8.**
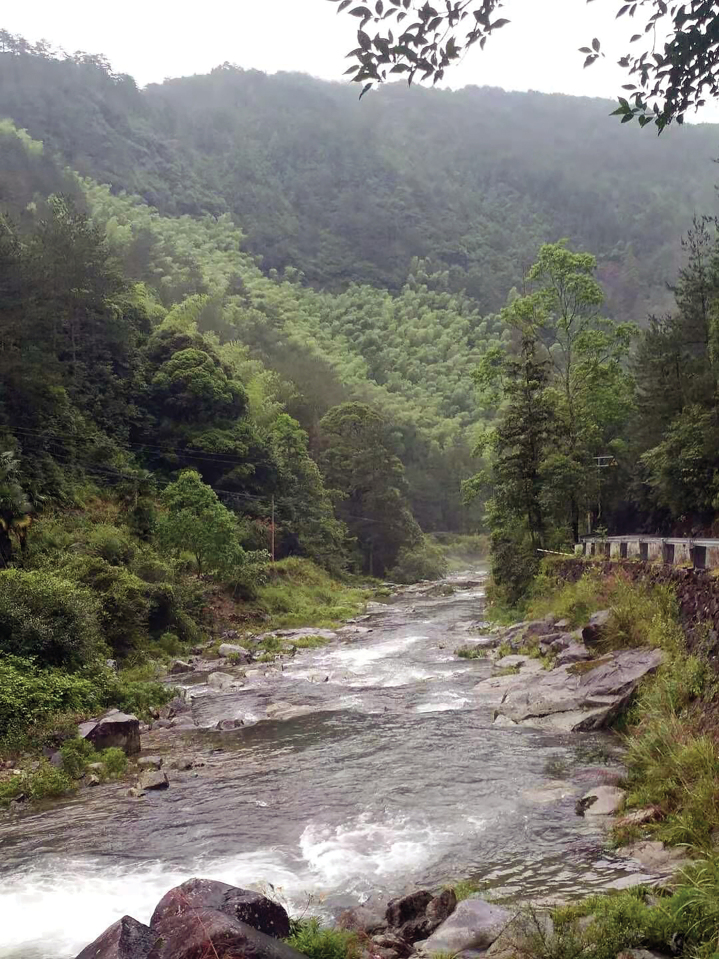
Habitat of *Sinocamaenacheni* Wu, gen. et sp. nov., Zhangjiadi, Yunhe County, Lishui.

## Supplementary Material

XML Treatment for Sinocamaena

XML Treatment for Sinocamaena cheni

## ﻿Additional information

### Author ORCIDs

Min Wu https://orcid.org/0000-0000-0000-0000

### Data availability

All of the data that support the findings of this study are available in the main text.

## References

[B1] Benson WH (1842) Mollusca. In: Cantor T (Ed.) General features of Chusan, with remarks on the flora and fauna of that island.The Annals and Magazine of Natural History, London 9: 486–490. 10.1080/03745484209445368

[B2] Capella-Gutiérrez S Silla-Martínez JM Gabaldón T (2009) trimAl: A tool for automated alignment trimming in large-scale phylogenetic analyses. Bioinformatics 25(15): 1972–1973. 10.1093/bioinformatics/btp348 19505945 PMC2712344

[B3] Chen Z-Y Lyu Z-T Wu M (2021) Systematic revision of *Stegodera* Martens, 1876 (Gastropoda, Stylommatophora, Camaenidae), with description of a new genus. ZooKeys 1059: 1–21. 10.3897/zookeys.1059.68385 34566444 PMC8426314

[B4] Gredler PV (1878) Zur Conchylien-Fauna von China. I. Stück Nachrichtsblatt der deutschen Malakozoologischen Gesellschaft 7: 101–105.

[B5] Gredler PV (1881) Zur Conchylien-Fauna von China. III. Stück Jahrbücher der Deutschen Malakozoologischen Gesellschaft 8: 110–132.

[B6] Gredler PV (1884) Zur Conchylien-Fauna von China. V. Stück Jahrbücher der Deutschen Malakozoologischen Gesellschaft 11: 129–161.

[B7] Gude GK (1902a) A classified list of the helicoid land shells of Asia. (Part II) i. The Chinese Empire. The Journal of Malacology 9: 51–59.

[B8] Gude GK (1902b) A classified list of the helicoid land shells of Asia (Part III) ii. Asiatic Russia. The Journal of Malacology 9: 97–129.

[B9] Gude GK (1902c) A Classified list of the helicoid land shells of Asia. The Journal of Malacology 9: 1–11.

[B10] Guindon S Dufayard JF Lefort V Anisimova M Hordijk W Gascuel O (2010) New algorithms and methods to estimate maximum-likelihood phylogenies: Assessing the performance of PhyML 3.0. Systematic Biology 59(3): 307–321. 10.1093/sysbio/syq010 20525638

[B11] Habe T (1945) Anatomical study of four snail species from Eastern Asia. Japanese Journal of Malacology 14(1–4): 14–21.

[B12] Henríquez FC Ibáñez M Alonso R (1993) Revision of the genus *Napaeus* Albers, 1850 (GastropodaPulmonata: Enidae). The problem of Napaeus (Napaeinus) nanodes (Shuttleworth, 1852) and description of five new species from its conchological group. The Journal of Molluscan Studies 59(2): 147–163. 10.1093/mollus/59.2.147

[B13] Heude PM (1882) Notes sur les mollusques terrestres de la vallée du Fleuve Bleu. Mémoires concernant l’histoire naturelle de l’Empire chinois 1: 1–84. 10.5962/bhl.title.50365

[B14] Heude PM (1885) Notes sur les mollusques terrestres de la vallée du Fleuve Bleu. Mémoires concernant l’histoire naturelle de l’Empire chinois 2: 89–132.

[B15] Hwang C-C Zhou W-C Ger M-J Guo Y Qian Z-X Wang Y-C Tsai C-L Wu S-P (2021) Biogeography of land snail genus *Acusta* (Gastropoda: Camaenidae): Diversification on East Asian islands. Molecular Phylogenetics and Evolution 155: 106999. [Epub2020Oct30] 10.1016/j.ympev.2020.106999 33130300

[B16] Jones KH Preston HB (1904) List of Mollusca collected during the commission of H. M. S. “Waterwitch” in the China Seas, 1900–1903, with descriptions of new species. Proceedings of the Malacological Society, London 6: 138–151. 10.1093/oxfordjournals.mollus.a066053

[B17] Kalyaanamoorthy S Minh BQ Wong TKF von Haeseler A Jermiin LS (2017) ModelFinder: Fast model selection for accurate phylogenetic estimates. Nature Methods 14(6): 587–589. 10.1038/nmeth.4285 28481363 PMC5453245

[B18] Katoh K Standley DM (2013) MAFFT Multiple Sequence Alignment Software Version 7: Improvements in performance and usability. Molecular Biology and Evolution 30(4): 772–780. 10.1093/molbev/mst010 23329690 PMC3603318

[B19] Kerney MP Cameron RAD (1979) A Field Guide to the Land Snails of Britain and North-West Europe. Collins, London, 288 pp. [24 pls]

[B20] Kuroda T Habe T (1949) Helicacea. 6 + 129 pp., 1 pl. Sanmeisha, Tokyo. [in Japanese]

[B21] Martens EV (1873) Neue *Helix*-Arten aus China. Malakozoologische Blätter 21: 67–69.

[B22] Minh BQ Nguyen MAT von Haeseler A (2013) Ultrafast approximation for phylogenetic bootstrap. Molecular Biology and Evolution 30(5): 1188–1195. 10.1093/molbev/mst024 23418397 PMC3670741

[B23] Möllendorff OF (1874) Diagnosen neuer Arten aus dem Binnenlande von China. Jahrbücher der Deutschen Malakozoologischen Gesellschaft 2: 78–80.

[B24] Möllendorff OF (1875) Chinesische Landschnecken. Jahrbücher der Deutschen Malakozoologischen Gesellschaft 2: 118–135.

[B25] Möllendorff OF (1884) Materialien zur Fauna von China. Jahrbücher der Deutschen Malakozoologischen Gesellschaft 11: 307–391.

[B26] Möllendorff OF (1899) Binnen-Mollusken aus Westchina und Centralasien. I. Annuaire du Musée Zoologique de l’Académie Impériale des St.-Pétersburg 4: 46–144 [pls 2–8]. 10.5962/bhl.title.13125

[B27] Nguyen L-T Schmidt HA von Haeseler A Minh BQ (2015) IQ-TREE: A fast and effective stochastic algorithm for estimating Maximum-Likelihood phylogenies. Molecular Biology and Evolution 32(1): 268–274. 10.1093/molbev/msu300 25371430 PMC4271533

[B28] Páll-Gergely B Gojšina V Neubert E (2023) Revision of Burmochloritis Godwin-Austen, 1920 in Southeast Asia (Gastropoda: Stylommatophora: Camaenidae). Archiv für Molluskenkunde 152(2): 183–216. 10.1127/arch.moll/152/183-216

[B29] Palumbi S Martin A Romano S McMillan WO Stice L Grabowwski G (1991) The Simple Fool’s Guide to PCR. Department of Zoology, University of Hawaii, Honolulu.

[B30] Pfeiffer L (1859) Descriptions of twenty-seven new species of land-shells, from the collection of H. Cuming, Esq. Proceedings of the Zoological Society of London 27: 23–29. [pls. XLIII.–XLIV.]

[B31] Pilsbry HA (1887) In: GW Tryon and HA Pilsbry, Manual of Conchology (2) 3, 1–313 [63 pls].

[B32] Pilsbry HA (1888) In: GW Tryon and HA Pilsbry, Manual of Conchology (2) 4, 1–296 [69 pls].

[B33] Pilsbry HA (1890) In: GW Tryon and HA Pilsbry, Manual of Conchology (2) 6, 1–324 [69 pls].

[B34] Pilsbry HA (1892) In: GW Tryon and HA Pilsbry, Manual of Conchology (2) 8, 1–314 [58 pls].

[B35] Pilsbry HA (1934) Zoological results of the Dolan West China expedition of 1931, Part II, Mollusks. Proceeding of the Academy of Natural Sciences of Philadelphia 86: 5–28 [6 pls]

[B36] Pilsbry HA Hirase Y (1905) Catalogue of the land and fresh-water Mollusca of Taiwan (Formosa), with descriptions of new species. Proceedings. Academy of Natural Sciences of Philadelphia 57: 720–752.

[B37] Pilsbry HA Hirase Y (1908) New land shells of the Chinese Empire: I. Proceedings. Academy of Natural Sciences of Philadelphia 60(1): 37–43.

[B38] Ronquist F Teslenko M Van Der Mark P Ayres DL Darling A Höhna S Larget B Liu L Suchard MA Huelsenbeck JP (2012) MrBayes 3.2: Efficient Bayesian phylogenetic inference and model choice across a large model space. Systematic Biology 61(3): 539–542. 10.1093/sysbio/sys029 22357727 PMC3329765

[B39] Schileyko AA (2003) Treatise on recent terrestrial pulmonate molluscs. Part 11. Trigonochlamydidae, Papillodermidae, Vitrinidae, Limacidae, Bielziidae, Agriolimacidae, Boettgerillidae, Camaenidae. Ruthenica (Supplement 2): 1467–1626.

[B40] Wade CM Mordan PB (2000) Evolution within the gastropod molluscs; using the ribosomal RNA gene-cluster as an indicator of phylogenetic relationships. The Journal of Molluscan Studies 66(4): 565–570. 10.1093/mollus/66.4.565

[B41] Wang P Xiao Q Zhou W-C Hwang C-C (2014) Revision of three camaenid and one bradybaenid species (Gastropoda, Stylommatophora) from China based on morphological and molecular data, with description of a new bradybaenid subspecies from Inner Mongolia, China. ZooKeys 372: 1–16. 10.3897/zookeys.372.6581 PMC3909801 24493955

[B42] Wu M (2019) A taxonomic note on the helicoid land snail genus *Traumatophora* (Eupulmonata, Camaenidae). ZooKeys 835: 139–152. 10.3897/zookeys.835.32697 31046027 PMC6477829

[B43] Wu M (2023) A new *Aegistohadra* (Gastropoda: Camaenidae) from southwest China. Ruthenica 33(2): 59–71. 10.35885/ruthenica.2023.33(2).2

[B44] Wu M Asami T (2017) Taxonomical notes on Chinese camaenids with description of three new species (Gastropoda: Pulmonata). Molluscan Research 38(2): 137–148. 10.1080/13235818.2017.1380145

[B45] Wu M Qi G (2006) A taxonomic note on *Pseudiberus* Ancey, 1887 (Gastropoda: Pulmonata: Bradybaenidae). Folia Malacologica 14(1): 25–30. 10.12657/folmal.014.003

[B46] Wu M Chen Z-Y Zhang L (2019a) Jawless land snail *Sinorachis*, a new bradybaenine genus from China (Eupulmonata, Camaenidae). ZooKeys 853: 51–67. 10.3897/zookeys.893.38445 PMC6901612 31845925

[B47] Wu M Chen Z-Y Zhu X-R (2019b) Two new camaenid land snails (Eupulmonata) from Central China. ZooKeys 861: 129–144. 10.3897/zookeys.861.35430 31335921 PMC6629712

[B48] Wu M Shen W Chen Z-G (2023) Land snail diversity in central China: Revision of *Laeocathaica* Möllendorff, 1899 (Gastropoda, Camaenidae), with descriptions of seven new species. ZooKeys 1154: 49–147. 10.3897/zookeys.1154.86237 37251698 PMC10209311

[B49] Xia X (2018) DAMBE7: New and improved tools for data analysis in molecular biology and evolution. Molecular Biology and Evolution 35(6): 1550–1552. 10.1093/molbev/msy073 29669107 PMC5967572

[B50] Xiang C-Y Gao F Jakovlić I Lei H-P Hu Y Zhang H Zou H Wang G-T Zhang D (2023) Using PhyloSuite for molecular phylogeny and tree-based analyses. iMeta 2(1): e87. 10.1002/imt2.87 PMC10989932 38868339

[B51] Yen T-C (1935) The Non-marine gastropods of North China. Part I. Publications du Musée Hoangho Paiho de Tien Tsin No. 34: 1–57 [5 pls].

[B52] Yen T-C (1936) Notes on the distribution of non-marine mollusks in the Soochow area. China Journal (Canberra, A.C.T.) 24(1): 44–47.

[B53] Yen T-C (1938) Notes on the gastropod fauna of Szechwan Province. Sonderabdruck aus: Mitteilungen aus dem Zoolog. Museum in Berlin 23(2): 438–457 [1 pl.].

[B54] Yen T-C (1939) Die chinesischen Land- und Süßwasser-Gastropoden des Natur-Museums Senckenberg. Abhandlungen der Senckenbergischen Naturforschenden Gesellschaft 444: 1–234 [16 pls].

[B55] Yen T-C (1942) A Review of Chinese Gastropods in the British Museum. Proceedings of the Malacological Society of London 24: 170–288 [pl. 11–28].

[B56] Yen T-C (1943) Review and summary of Tertiary and Quaternary non-marine mollusks of China. Proceedings. Academy of Natural Sciences of Philadelphia 95: 267–309, 346.

[B57] Yen T-C (1948) Notes on land and fresh-water mollusks of Chekiang Province, China. Proceedings of the California Academy of Sciences Fourth Series 24(4): 69–99 [1 pl.].

[B58] Zhang L-J Zhu Y-J Lyu Z-T (2019) A new sinistral species of the land-snail genus *Satsuma* (Pulmonata: Camaenidae) from China. Molluscan Research. 10.1080/13235818.2019.1644721

[B59] Zhang D Gao F Jakovlić I Zou H Zhang J Li WX Wang GT (2020) PhyloSuite: An integrated and scalable desktop platform for streamlined molecular sequence data management and evolutionary phylogenetics studies. Molecular Ecology Resources 20(1): 348–355. 10.1111/1755-0998.13096 31599058

[B60] Zhang G-Y Wu M Köhler F Liu T-T (2021) Review of the genus *Pseudiberus* Ancey, 1887 (Eupulmonata: Camaenidae) in Shandong Province, China. Malacologia 63(2): 257–284. 10.4002/040.063.0207

[B61] Zhou W-C Xiao Q Chen D-N Hwang C-C (2011) *Plectotropisyonganensis* sp. nov. (Gastropoda: Bradybaenidae) from China, with revision of two Chinese camaenid species (Gastropoda: Camaenidae). Zootaxa 2929(1): 51–56. 10.11646/zootaxa.2929.1.4

[B62] Zilch A (1968) Die Typen und Typoide des Natur-Museums Senckenberg. 41. Archiv für Molluskenkunde 98(3/4): 155–212.

[B63] Zilch A (1974) Vinzenz Gredler und die Erforschung der Weichtiere Chinas durch Franziskaner aus Tirol. Archiv für Molluskenkunde 104(4/6): 171–228 [pls. 7–9].

